# Discussing the Effect of Students' Crisis Awareness on Emotion During the COVID-19 Pandemic From the Perspective of Trust

**DOI:** 10.3389/fpsyg.2022.803372

**Published:** 2022-07-04

**Authors:** Cheng Yang, Yinghua Miao

**Affiliations:** ^1^School of Business Administration, Nanchang Institute of Technology, Nanchang, China; ^2^Network Information Center, Nanchang Institute of Technology, Nanchang, China

**Keywords:** coronavirus disease 2019, crisis awareness, trust, emotion, time pressure

## Abstract

The effects of crises vary among individuals, societies, and nations. Governments' crisis management is quite different from that of non-governmental organizations, especially in terms of “publicity,” since it involves bureaucracy to address people's accountability concerns. The purpose of this study is to investigate the relationship between students' crisis awareness, trust, and emotions in the event of a major public health emergency. A questionnaire survey was conducted for this study. A total of 500 copies of questionnaires were distributed to the college students in Jiangxi. Among those, 437 valid copies were retrieved, with a retrieval rate of 87%. A structural equation model (SEM) was used to conduct the statistical analyses. The research results were summarized as follows: (1) At the stage of epidemic spread, people can easily fall into the negative emotion. (2) The society with a good trust relationship considers schools less responsible for critical incidents and more helpful for crisis communication. (3) Reducing the negative emotions of the public after the occurrence of critical incidents can effectively reduce the damage of critical incidents to the organization. Avoiding a loss of student confidence and increasing anger, protecting the school's reputation, having a good communication effect, and minimizing the impact of the crisis can help the students develop better trust toward the school. When a crisis occurs on campus, this can reduce the possibility of students' showing negative emotions and spreading rumors. It is considered that the findings provide guidance on how to optimize the management of public health crisis situations and improve students' mental health.

## Introduction

It is inevitable that public institutions, private business enterprises, or non-governmental organizations will face various forms of crisis situations. None of these organizations is able to escape a crisis. Even organizations with a good reputation are exposed to the risk of crises. This is the time of crisis in society when individuals in all organizations with external interaction can experience the severe conditions of crisis. The effects of crises are different for individuals, society, and the nation. When crisis events affect the safety of people and property, they attract attention, and the injuries and losses suffered can more easily attract attention. A media report about an accident or disaster can easily draw public attention to the issue. Similar to diseases, the development of the Internet in recent years has accelerated the flow of information in society. The media can quickly update the news network to instantly spread crisis events throughout the world (Huang and Chen, 2020).

The great epidemic of novel coronavirus pneumonia in 2020 was a test for the world. By March 29, 2021, there were more than 127 million confirmed cases worldwide. The World Health Organization (WHO) announced that it had classified the epidemic as a public health incident. Crises are incidents with high uncertainty and threatening aspects. As in the example of diseases, crises show the stages of incubation, outbreak, sequelae, and resolution. The development of true crises can be irregular and repetitive, similar to diseases. The outbreak does not occur for no reason; it could be an infected virus that has been in incubation for a period of time (Chan et al., 2020). With proper treatment during this period, it would recover without an outbreak. Effective crisis management is important for crises. Crisis management can be divided into different aspects and phases, such as the phases before, during, and after the incident, or precaution, preparedness, response, and recovery. The phases of learning and evaluation can also be included at the end. Different experts work on different topics and objects and study crisis management from different aspects. Such management also includes many processes such as planning, organization, budget, and control. However, there exist great differences in crisis management between governmental and non-governmental organizations, especially in terms of “publicity” since it involves bureaucracy to address people's concerns about accountability.

Since the outbreak of COVID-19, schools are moving to online courses in various areas. Huang and Chen (2020) conducted a study with the participation of professors in Hong Kong to study the effects of novel coronaviruses on students and office workers worldwide. They discovered that 48% of the respondents noted an increase in study stress and 54% of them reported a decrease in learning efficiency due to web-based learning. Compared to students, office workers generally accepted and satisfied with the home-based work arrangement. The survey on HK employees' opinions and experiences of working from home revealed that more than 70% of the interviewees considered that they had more resting time in home-based work, 64% reported a decrease in working pressure, and about 50% (49.2%) revealed that home-based work led to better family relationships. Clearly, coronavirus disease had stronger emotional effects on students than on office workers. Li et al. (2020) analyzed the factors for students' academic success and found out that 31% of the factors were due to positive emotions, including hope, wellbeing, and engagement. The survey revealed the highest percentage for hope (13%), followed by engagement (10%) and wellbeing (8%). Olivia Schultes et al. (2021) pointed out the importance of having positive emotions to successfully navigate a new environment, build meaningful relationships, and reach a person's maximum potential. Huang and Chen (2020) mentioned that teachers' emotional awareness and unconditional positive concerns can predict students' adaptation to schools, trust, and emotions.

Chan et al. (2020) mentioned that the British Academy collected 583 copies of questionnaires from international/local college students in 26 countries and regions in Africa, Asia, Australia, Europe, North America, and South America. It was found out that up to 90% of the interviewees revealed “medium to great” effects of the epidemic on the learning activity. During the survey, 61% of the interviewees were in the countries and regions where they studied, and 14% of them did not know where to seek for medical assistance when symptoms appeared. Moreover, about half of the interviewees (47.5%) thought about the risk of confirmed coronavirus disease, and about 71% of them showed anxiety. This awareness of crisis and epidemic prevention measures, such as reducing social contact, resulted in loneliness in 45% of the interviewees.

Mouter et al. (2021) stated that in times when society faces great environmental pressure, people may panic due to inadequate information and worry about other people's views, the possible effects on their lives, and ignorance of the epidemic, leading to a fear of confirmation. In addition, it was normal to be nervous, anxious, and uncertain about symptoms and consequences when they have a confirmed diagnosis. Klapperich et al. (2020) mentioned that the outbreak of the COVID-19 epidemic resulted in more people having to stay at home and self-monitor their health. In addition to readjusting to their lives and work, they suffered from tremendous physical and psychological stress during the epidemic. Clinically, many people needed psychotherapy because they were too preoccupied with the intense media coverage of the growing epidemic. Lin et al. (2020) indicated that many students were taking online courses and living with their parents because of the epidemic. The friction with parents during the epidemic resulted in great perceived pressure; those with suppressed personalities could be easily depressed, did not know how to release their emotions, could not focus on online courses, and were easily irritable. The pressure exceeded the critical point of self-adjustment and could not be managed, so the cases of psychiatric/psychological health diagnoses caused by coronavirus disease increased greatly.

With the rapid change in the social environment, crises such as natural disasters, accidents, human factors, and suicide keep occurring on the campus, which was originally a safe and happy place of learning. Each event is in the media spotlight (Rezaei et al., 2014). In 2005, in order to maintain campus safety, Lee (2016) divided campus events into “campus accident,” “maintaining campus safety,” “campus violence and deviant behavior,” “discipline controversy,” “protection of children and youth,” “natural disaster,” “other campus affairs,” and “illness.” According to the statistics, illnesses occurred most frequently, followed by accidents on campus. Illnesses cannot be controlled by school administrators, but accidents are not completely unavoidable; most accidents could be prevented. Schools should take responsibility for the resurgence of the local COVID-19 epidemic in China and the spread on campus. The epidemic in China is currently showing several outbreaks. Considering the prevention and control of the current epidemic situation, many schools have decided to strengthen the daily management of students (Chan et al., 2020). All students stay in school, and the process of learning continues in school. Olivia Schultes et al. (2021) found that more frequent testing would increase the case rate but could better prevent the spread of infection. They also mentioned that campuses are not closed groups, so even if there is a vaccine, schools should continue testing students for COVID-19 and implement strategies to slow the spread of infection. Clearly, crisis awareness among both schools and students is extremely important.

Most previous studies on the crisis have addressed crisis management issues. In this study, it was found that data analysis was rarely used in previous studies. On the contrary, qualitative research or content analysis was mostly used (Samantha, 2018; Eggers, 2020; Fabeil et al., 2020). Moreover, the core of the crisis focused on the relevance of the “situation-strategy-effect,” and the communication effect was mostly tested with a single situation and a single strategy, while the effects on the affective states of research participants' were rarely discussed (Huang and Chen, 2020; Vally, 2020; Mouter et al., 2021). Regardless of whether the participants were from the public sector, private companies, or non-profit organizations, the crisis of campus students was rarely considered as a research direction. This study aims to discuss the relationships between student crisis awareness, trust, and emotions during major public healthcare emergencies to further optimize crisis management and improve students' mental health.

## Literature Review and Hypotheses

### Research on Crisis Awareness and Trust

Vally (2020) defined a crisis as an unforeseen crisis in an organization for which there are no programs or plans. Cheng and Hahm (2019) defined a crisis as a disruption to the overall operation of the system and a threat to the basic setting, self-subjective perception, and current core objectives. Janssens et al. (2019) considered that crisis is used in conjunction with the meanings of threat or dilemma; crisis refers to the individual's or group's perception of potential negative effects that result from not applying certain remedial actions. Akgunduz and Eryilmaz (2018) considered that a crisis seriously threatens the basic gains and structure of a society, system, or organization and even threatens fundamental values and norms. Bansal et al. (2020) classified the characteristics of a crisis as uncertainty, incomplete information, communication difficulties, and complicated conditions. From a management perspective, people in a crisis have to make important decisions under time pressure and in an uncertain situation.

Lin et al. (2020) considered external communication at the first moment as the key to the success of crisis management rather than the whole crisis management plan. This is because a crisis would raise the crisis awareness of the media and the public. Without developing appropriate communication strategies, this could easily affect trust in the organization, leading to an outcry and even a boycott. The impact on the organization is therefore obvious. The epidemic, according to Chan et al. (2020), highlighted the importance of transparency in rebuilding trust for solutions in times of trust crisis. They found out that trust in the government was not the only important issue but that society considered different levels of trust given the impact of the epidemic, especially trust in civil society, public health experts, and the government, and the mutual effect between these variables. Trust was essential to stop the virus in time. Flexible and sustainable long-term policies, as well as transparency and openness of information, were extremely important. Research revealed that transparent information was the best way for the government to influence public awareness of the crisis awareness and rebuild trust during the epidemic, and that information should be clear and consistent. Forster et al. (2020) believe that the relationship between crises and emotions is the reason why the public is affected by crisis events that trigger emotions that are detrimental to both the organization and public trust. The public or interested party would evaluate an organization's responsibility for crisis events through attribution. The emotions of the public are influenced by the attribution of responsibility. The higher the attribution of responsibility, the greater the public anger, which affects the trust in the organization. In this case, the damage that crisis events inflict on an organization and the extent to which they elicit negative public trust and emotions were defined by public crisis awareness. Vojtko et al. (2019) stated that mass society, after receiving crisis information from the media, does not immediately look for the cause of the incident but may reduce trust and emotions based on the outcome of the incident. The resulting emotions were derived from the incident process after receiving the media report. In this case, this study proposes the following hypothesis:

**Hypothesis 1**: Crisis awareness has a negative and significant impact on trust.

### Research on Trust and Emotions

Research on trust has been discussed in various fields, such as psychology, sociology, economics, and marketing. Fritz and Gallagher (2020) defined “trust” as a party willing and expecting the other party to perform a specific behavior without monitoring or controlling the other party in the process. According to the literature, trust is a necessary factor for two groups to join together because the existence of trust can reduce the risk of cooperating parties and guarantee future gains (Natarajan and Gombolay, 2020). In other words, if trust between two groups led them to believe that the opposite parties would fulfill the obligation, the risks caused by speculation could be reduced (Loffler et al., 2020). Thus, the trust between companies and customers is similar to the trust between people, which helps understand each other and has positive effects on the mutual relationship. In this case, a company would be welcomed by partners with capabilities, goodwill, and honesty to enhance the trust. Wang et al. (2020) considered that the trust relationship between consumers and companies should be taken into account when considering a crisis situation. The trust relationship between a company and an interested party offered the significant explanatory potential for the crisis situation. Benvenuto et al. (2020) indicated that consumers in crisis situations would try to analyze and perceive crisis situations from the perspective of companies. In other words, mass consumers analyze and judge crisis situations differently depending on the quality of their trust relationship with the brand. The quality of the trust relationship between consumers and a brand would influence and shape consumers' judgment and perceptions of crisis situations and affect their opinions about the enterprise during the crisis. A poor trust relationship or crisis background would make it easier for the public to attribute responsibility for the crisis to the companies and show their emotions. Pavlatos and Kostakis (2018) mentioned that the public would attribute less responsibility to companies with a good trust relationship during a crisis incident in order to generate less negative emotions toward the companies. Mouter et al. (2021) stated that people's trust in the government affects their attitude toward epidemics. In other words, trust in government influences emotions to affect epidemic prevention behaviors; people who believe in the latest and correct epidemic information provided by government agencies show positive emotional perceptions to enhance the effect of epidemic prevention. Therefore, this study proposes the following hypothesis:

**Hypothesis 2**: Trust has positive and significant effects on emotions.

### Research on Crisis Awareness and Emotion

Emotions, complicated psychological processes, are triggered by certain events in the external environment (Wang et al., 2019). Klapperich et al. (2020) defined emotion as an individual psychological response to stimuli in the external environment to acquire subjective emotions and individual experiences. Roy et al. (2019) considered that emotion is psychologically complicated and influences physiological response, which includes individual subjective mental state, individual impulse to take action, and individual major physical changes. Michelle et al. (2019) proposed emotion as a natural emotional response to the interpretation of events. In psychology, emotion refers to the individual physiological response affected by stimuli in the external environment or internal physical conditions to cause an individual psychological imbalance. The imbalanced state was chaotic, agitated, excited, and nervous state.

Huang et al. (2020) explained emotions as psychological reactions triggered by the external environment or specific events that elicit different emotional responses and lead to different behavioral intentions. The research on crisis communication rarely focused on the effects of emotional reactions of the interested party and the society to crisis events in organizations. However, dealing with the emotions of the interested party and society as a whole is an important part of crisis events. When society shows dissatisfaction toward an organization, the organization's crisis response strategies are reduced. Erokhin et al. (2019) pointed out the mediating role of public anger between crisis responsibility and negative image and suggested that an organization should consider reducing public anger as the primary goal when managing crisis events to reduce the impact of negative rumors caused by the public anger on the organizational image. Schupper et al. (2020) found out that of the many emotions felt by the public during an incident, only anger and vigilance have significant effects on the organizational image. In this case, after the occurrence of crisis events, an organization must first assess the emotion type of the interested party to respond to the various emotions of the public. Reducing the emotional response of the public can effectively reduce the damage of crisis events to the organization. Fabeil et al. (2020) pointed out the difficulty of effectively building a favorable communication bridge with the public during the epidemic. People would panic and become aware of the crisis because they do not understand the government's epidemic prevention measures to show further present negative emotions without effective cooperation to reduce the epidemic prevention effect. Accordingly, this study proposes the following hypothesis:

**Hypothesis 3**: Crisis awareness has a negative impact on emotions.

## Methodology

### Conceptual Structure

**Figure 1:**
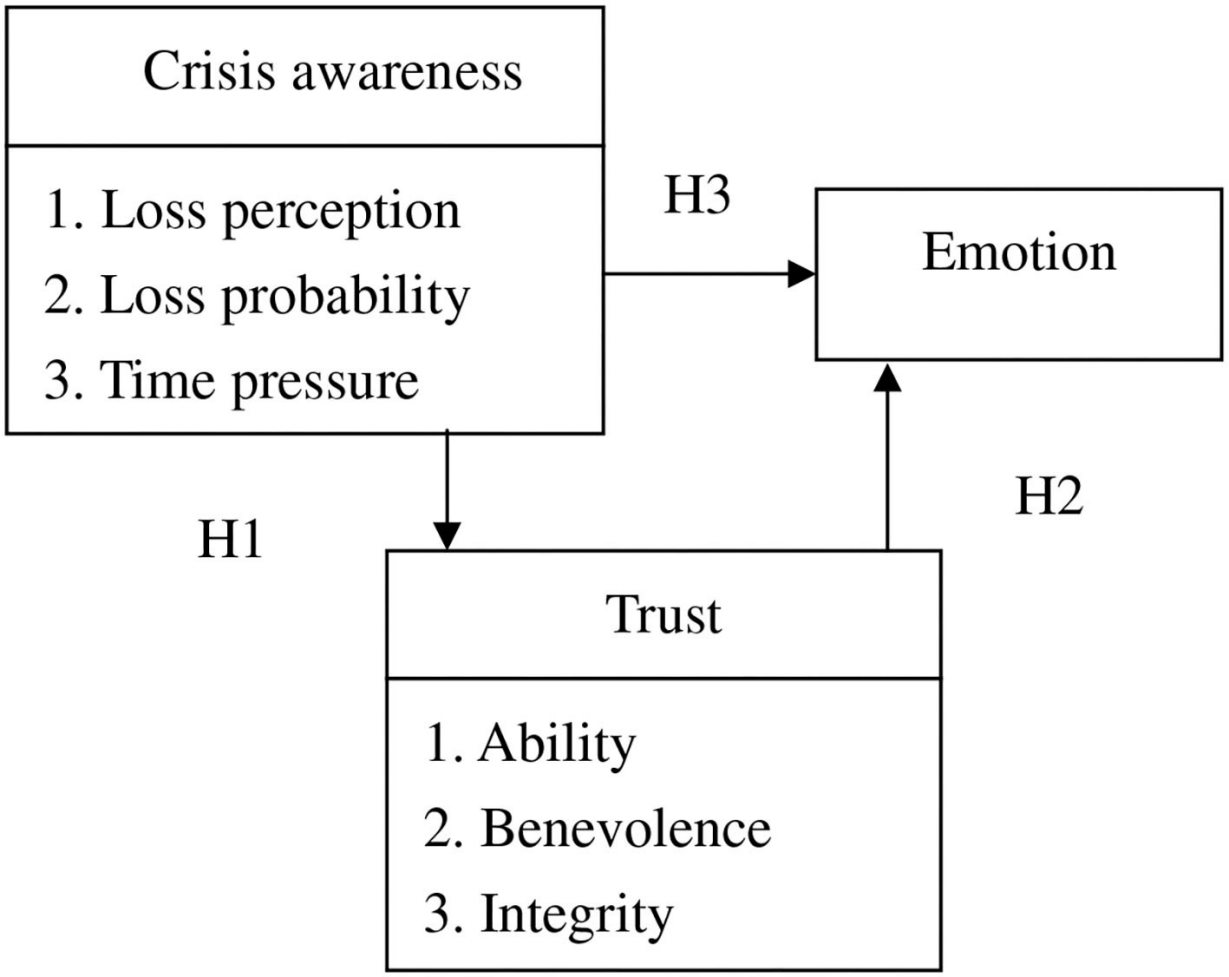
Conceptual structure.

Based on the literature review above, the conceptual structure for this study was designed (Figure 1) to investigate the relationships between crisis awareness, trust, and emotions.

### Operational Definition

#### Crisis Awareness

Crisis awareness as an independent variable in this study contains three dimensions, according to the blended learning model proposed by Zhang et al. (2020).

1. Loss perception: The difference between the actual and ideal conditions could be the determinant of possible loss of value. But the importance of the problem could influence the perception of lost value. That is, the same crisis could lead to different losses in different organizations, even if the problem is the same.
2. Loss probability: Even though the potential loss of value is high, the low probability of occurrence may reduce the perception of crises.
3. Time pressure: The more distant the time of the possible negative outcome, the less the crisis can be perceived. Among the factors involved in the perception of time as a source of pressure is that 1. It becomes serious if the problem is not solved. In other words, if the problems are not solved, the negative effects may be perceived more strongly, and 2. The time to find appropriate solutions is limited.

#### Trust

In terms of trust, as the dependent variable in this study, following the trust model proposed by Huang et al. (2021), three dimensions are organized for building trust in this study.

1. Ability: This refers to individuals or enterprises that are preferred by trustees because they have rich knowledge in specialized or professional fields that meet the needs of trustees (clients). At the same time, the trustees also believe that the trusted individuals or enterprises are capable of efficiently fulfilling their promises.
2. Benevolence: This refers to individuals or companies that have special feelings for the trustees and care about them from the heart. Individuals or enterprises with benevolence assume the sustainability of clients' equity and even sacrifice equity to maintain clients' profits.
3. Integrity: This refers to individuals or enterprises that abide by the agreement with the trustees under their care, without destroying or disregarding certain promised principles.

#### Emotion

The emotion scale, as the dependent variable in this study, was used to measure individuals' emotions during the pandemic, according to the emotion model proposed by Li et al. (2020).

### Participants and Objectives

The participants of the study were composed of college students in Jiangxi Province. Convenience sampling was adopted in this study. The school addresses and contact persons were first confirmed to explain the research objective and request. After receiving the consent to cooperate, the folders, containing the questionnaire, the printout of precautions, the envelope, and a nice gift, were sent to the schools personally.

To ensure that the samples match the population proportion, case officers were verbally informed of the arrangements made by the researcher for distributing the questionnaires. These included samples that cover all genders, grades, and departments were distributed on average and do not focus on a single grade or division. The written arrangements and the questionnaire were then sent to the schools in the sample. A total of 500 copies of the questionnaire were distributed. Among those, 437 valid copies were retrieved with a retrieval rate of 87%. The sample structure of this study contains 223 males and 214 females. Among the participants, the participants' distribution by their level of education was determined as 108 first-year students, 106 second-year students, 112 third-year students, and 111 fourth-year students. The participants were composed of 74 students from North China, 122 students from East China, 83 students from Central China, 60 students from South China, 67 students from Southwest China, and 31 students from Northwest China. The family economic status of students was found as 72 wealthy, 289 well-off, 47 poor, and 29 low-income families.

### Reliability and Validity Test

Confirmatory factor analysis (CFA) is an important part of SEM. The measurement model should be tested before modifying the structural model evaluation with the two-level model. When the goodness-of-fit of the measurement model is acceptable, SEM is conducted as the second step. The dimensions analyzed in this study with CFA have the factor loadings between 0.60 and 0.90, the composite reliability between 0.80 and 0.90, and the average variance extracted between 0.70 and 0.85. These results meet the standards of 1. factor loadings >0.5, 2. composite reliability >0.6, and 3. average variance extracted >0.5. Thus, the dimensions exhibit present convergent validity.

### Correlation Analysis

Pearson correlation analysis is used to examine the linear correlations between two continuous variables (X, Y). A large absolute correlation coefficient between these two variables indicates a large covariance. When two variables have a positive correlation, Y generally increases as X increases. In contrast, Y decreases with increasing X, if there is a negative correlation between the two.

### Structural Equation Modeling

Structural equation modeling (SEM) is a method for dealing with the measurement error. It uses multiple indicators to reflect the latent variables and makes the estimation between model factors more accurate and reasonable than the traditional regression method. Structural equation modeling and covariance structure modeling (LISREL) are popular and important data analysis skills. In the research for higher degrees in universities, it is an important topic for multivariate analysis; comparatively important social, educational, and psychological journals have special sections for it. Obviously, the fame and high status of SEM in statistics are undoubted.

## Results

### Structural Model Analysis

The structural model analysis includes the goodness-of-fit analysis of the research model and the explanatory power of the whole research model. Thus, referring to experts' opinions, seven numerical indices were used to test the overall model fit, including the chi-square (χ^2^) test, the χ^2^-degree of freedom ratio, the goodness-of-fit index, the adjusted goodness-of-fit index, the root-mean-square error, the comparative fit index, the comparative hypothesis model, and the chi-square test of independence. The overall results of the analyses were summarized in Table 1.

**Table 1:** Goodness-of-fit analysis of the research model.

**(Fit Indices)**	**Allowable**	**This research**	**Model fit**
	**range**	**model**	**judgment**
χ^2^ (Chi-square)	The lower the better	19.63	
χ^2^-degree of freedom ratio	<3	1.83	Match
GFI	>0.9	0.95	Match
AGFI	>0.8	0.89	Match
RMSEA	<0.08	0.04	Match
CFI	>0.9	0.94	Match
NFI	>0.9	0.92	Match

In summary, when testing the model fit with the χ^2^-degree of freedom ratio, the ratio should be lower. The χ^2^-degree of freedom ratio in this study was found to be <3 (1.83). GFI and AGFI are expected to be close to 1, and there are no absolute standards to judge the model fit. Moreover, the results for GFI and AGFI were found to be acceptable with GFI > 0.9 and AGFI > 0.8. GFI and AGFI in this study were 0.95 and 0.89, respectively. RMSEA in 0.05–0.08 indicates a good model with a reasonable fit. RMSEA in this study was found as 0.04. The allowable standard of CFI is > 0.9; and the CFI of the research model in this study was found as 0.94. The NFI should be at least higher than 0.9; and the NFI of this research model was found as 0.92. Overall, the goodness-of-fit indices meet the standards and indicate that the research results of the model are acceptable. Therefore, the research sample data can be used to explain the actual observed data.

The previous overall model fit indices have shown that there existed an acceptable goodness-of-fit between the model structured in this study and the observed data. These results indicate that the theoretical model can fully explain the observed data. In this case, the correlation coefficients and coefficient estimates of crisis awareness, trust, and emotion could be better understood after the model fit test.

The research data are given in Table 2. The results of the analysis of the whole model showed that three dimensions of crisis awareness (loss perception, loss probability, and time pressure) can significantly explain crisis awareness (*t* > 1.96, *p* < 0.05). Similarly, the three dimensions of trust (ability, benevolence, and integrity) can explain trust significantly (*t* > 1.96, *p* < 0.05). Obviously, the overall research model shows a good preliminary fit.

**Table 2:** Overall results of linear structural model analysis.

**Evaluation item**	**Parameter/evaluation standard**		**Result**
Preliminary fit	Crisis awareness	Loss perception	0.72^**^
		Loss probability	0.66^*^
		Time pressure	0.63^*^
	trust	Ability	0.75^**^
		Benevolence	0.69^*^
		Integrity	0.73^**^
Internal fit	Crisis awareness → trust		−0.83^***^
	Trust → emotion		0.82^***^
	Crisis awareness → emotion		−0.87^***^

* *p < 0.05*,

** *p < 0.01*,

*** *p < 0.001*.

In terms of internal fit, crisis awareness shows negative and significant correlations with trust (−0.83, *p* < 0.01), trust reveals positive and remarkable correlations with emotion (0.82, *p* < 0.01), and crisis awareness shows negative and notable correlations with emotion (−0.87, *p* < 0.01). Accordingly, it can be concluded that H1, H2, and H3 were supported.

### Correlation Analysis

The variables in the research structure were analyzed using Pearson correlation analysis as the preliminary test to discuss the correlations between the variables. The results are shown in Table 3.

**Table 3:** Overall correlation analysis.

**Variable**	**Loss perception**	**Loss probability**	**Time pressure**	**Ability**	**Benevolence**	**Integrity**	**Emotion**
Loss perception	–						
Loss probability	0.62**	–					
Time Pressure	0.66**	0.57**	–				
Ability	−0.71**	−0.69**	−0.72**	–			
Benevolence	−0.76**	−0.75**	−0.74**	0.51**	–		
Integrity	−0.79**	−0.72**	−0.77**	0.55**	0.57**	–	
Emotion	−0.81**	−0.86**	−0.82**	0.74**	0.70**	0.73**	–

### Analysis Result

The research results revealed the significance of the model. The results of the hypotheses tests are shown in Table 4.

**Table 4:** Hypotheses testing.

**Research hypotheses**	**Correlation**	**Empirical result**	***P***	**Result**
Hypothesis 1	–	−0.83***	0.00	Supported
Hypothesis 2	+	0.82***	0.00	Supported
Hypothesis 3	–	−0.87***	0.00	Supported

## Discussion

High negative emotions of students with high crisis awareness show a similar conclusion as the previous studies. Karkoulian et al. (2016) found that people with an internal locus of control attribution style may perceive greater pressure when faced with limited resources and time pressure. When faced with severe and unpredictable threatening events, individual efforts might not affect the result. Therefore, it might be better for individuals to control their attitudes. It is discovered in this study that students when faced with a serious threat of a pandemic, they were more concerned with the contemporary emergency factors mentioned above, while the psychological factor of trust, which requires rational thinking and comprehensive judgment, did not seem to be as important. With low crisis awareness, the rational person hypothesis develops the function when students' safety is not seriously affected by the pandemic. Similarly, people could make rational thinking and comprehensive analysis to show less-negative emotions. In this study, trust with new perspectives in a major public health incident has certain theoretical and practical meanings. First, the general public did not show high negative emotions triggered by the COVID-19 pandemic. However, they showed higher trust in the government in terms of effectively controlling the pandemic. This shows that the party and the government have the status in people's minds that the governing capacity is tested and recognized. Second, emotions are influenced by the environment. During the pandemic, people's negative emotions should be released and integrated through various channels. Finally, increasing the trust or decreasing people's perceived threat can positively influence people's physical and mental health when they face similar incidents in the future. Schools should take responsibility for campus accidents. Nevertheless, schools with limited budgets would not necessarily adopt a compensation strategy, instead, an apology, expression of concern, or correction of action can achieve a similar communication effect (Andreassen et al., 2016). It is suggested that schools should portray sincerity in problem-solving when using communication strategies to reinforce the communication effect and protect their reputation. When critical incidents occur on campus, the school should explain the cause as soon as possible and adopt a responsible attitude to reduce the students' awareness of the school's responsibility for crises, thus reducing students' anger and avoiding rumors. It can also facilitate the acceptance of explanations, protect the organization's reputation, and have a good communication effect. A school with a better reputation can reduce the possibility of negative word of mouth by students in the event of a crisis on campus. This proves that a good trust relationship can benefit the effect of crisis communication. In addition, school crisis managers should respond immediately to the outside world and not hide from the media when critical incidents occur on campus. Otherwise, there could be negative media exposure when the media asks for a third party's opinions, and the damage to the school from critical incidents will be even greater. The school's crisis managers should respond well to the problem and keep the emotions of stakeholders high during campus critical incidents. A gloat mainly comes from the public, which is composed of non-stakeholders. Crisis managers should respond to every part of a critical incident and avoid a gloat of public opinion in order not to damage the school's reputation. Trust depends on the public evaluation of the school's past performance. Once campus accidents occur, the school's communication would be more easily accepted to avoid emotional and negative word-of-mouth (Dowling and Doyle, 2017). For this reason, schools are recommended to abandon their closed-minded attitude, connect with people in the community, and even actively contact the media to establish a good with the public to improve their reputation.

## Conclusion

The study results revealed that certain rumors can exist in rural areas with limited information during the pandemic, especially at the beginning of the pandemic. People can easily feel negative emotions when there are no official information and people are caught off guard by the crisis. The higher the risks in crisis events, the stronger the negative emotions are experienced, as well as the more negative emotions can be observed to loop with rumors. Most students receive crisis information through video footage in the media. Therefore, school crisis managers need to reassure students who watch videos in the media and have high crisis awareness after the outbreak of COVID-19 to restore trust in schools and reduce students' intention to spread negative word of mouth. Moreover, finding crisis management methods and crisis communication content that reduce students' negative emotions can effectively mitigate the impact of crisis responsibility from the public's perspective. Furthermore, rural areas are places where people live in groups. Therefore, this lifestyle can easily lead to a cluster effect. Individual negative emotions caused by information overload can easily spread in these environments. This finding is similar to the results of the studies conducted during the SARS epidemic (Qian et al., 2003) and H7N9 avian influenza (Zhang et al., 2015). In a situation with a greater threat factor, people can perceive external risks more easily, and higher crisis awareness can easily result in negative emotions. Zhou et al. (2020) pointed out in their study that people who were more exposed during the SARS epidemic, such as healthcare workers in isolation wards, experienced significantly more psychological pain than other healthcare workers. In addition to the risk of exposure, fear of infection was the most important factor in the stress response. People with a high perception of crisis rated the likelihood of infection higher and were more concerned about their personal safety. It is therefore understandable that their negative emotions were higher than those of individuals with a low threat perception. Berg and Aber (2015) mentioned that in cases of campus accidents, the public's acceptance of the organization's perception of crisis responsibility, anger, and acceptance of the organization's explanation would be influenced by the reputation of the organization to generate negative word-of-mouth (Cavaiola and Colford, 2017). In other words, a school with better trust would reduce negative emotions and negative word-of-mouth from the public when there is a crisis on campus. This proves that the good reputation of the school would benefit the effect of crisis communication (Kar, 2014).

## Data Availability Statement

The original contributions presented in the study are included in the article/supplementary material, further inquiries can be directed to the corresponding author/s.

## Author Contributions

CY performed the initial analyses and wrote the manuscript. YM assisted in the data collection and data analysis. Both authors revised and approved the submitted version of the manuscript.

## Funding

This study was supported by the Humanities and Social Sciences program of the Ministry of Education (20YJC840036), Jiangxi University Humanities and Social Sciences Project (XL19106), Jiangxi Social Science Planning Project (20JY25), Education Planning project of Jiangxi Province (20YB210), and Jiangxi Instructor Master Studio Project.

## Conflict of Interest

The authors declare that the research was conducted in the absence of any commercial or financial relationships that could be construed as a potential conflict of interest.

## Publisher's Note

All claims expressed in this article are solely those of the authors and do not necessarily represent those of their affiliated organizations, or those of the publisher, the editors and the reviewers. Any product that may be evaluated in this article, or claim that may be made by its manufacturer, is not guaranteed or endorsed by the publisher.
